# Supplementary material for: Synergic versus Antagonist Effects of Rutin on Gallic Acid or Coumarin Incorporated into Chitosan Active Films: Impacts on Their Release Kinetics and Antioxidant Activity
Source: Antioxidants (Basel). 2023 Oct 30;12(11):1934. doi: 10.3390/antiox12111934 (PMC10669362; doi:10.3390/antiox12111934)
Supplement: Supplementary file 1 [file antioxidants-12-01934-s001.zip › antioxidants-2679134-supplementary.pdf]

Table S1: Physical-chemical parameters of selected antioxidants (data from www.ChemSpider.com).

Structure and physical-chemical parameters	Coumarin (Coum)	Gallic acid (GA)	Rutin (Rut)
Chemical structure	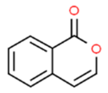	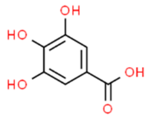	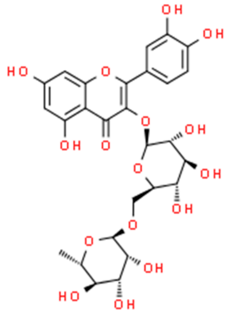
Chemical formula	$C_9H_6O_2$	$C_7H_6O_5$	$C_{27}H_{30}O_{16}$
Chemical name	1,2-Benzopyrone	3,4,5-Trihydroxybenzoic acid	3,3′,4′,5,7-Pentahydroxyflavone 3-rutinoside
CAS	91-64-5	149-91-7	153-18-4
Molecular weight ($g.mol^{-1}$)	146.43	170.12	610.51
Molar volume ($cm^3.mol^{-1}$)	193.2	97.3	n.d[1].
Density (ρ) ($g.cm^{-3}$)	0.935	1.694	1.80
Melting point MP (°C)	68-70	251	195
Boiling point BP (°C)	297-301	501	983.1
Solubility in water at 20°C ($g.L^{-1}$)	1.9	1.16	0.125
Pka in water or aqueous solution	4.5	3.94	6.17
Log P (water/octanol partition)	1.39	0.91	1.97

[1] n.d: not determined.

Figure S1: Release kinetics of each antioxidant compounds from the different film compositions: experi-mental data (symbols)and diffusion model fitting (lines) - Ct, concentration at time t, C∞, concentration at infinite time (at equilibrium).

<table>
<tr><th>Antioxidants</th><th>CH containing one antioxidant</th><th>CH-Rut+Coum</th><th>CH-Rut+GA</th></tr>
<tr><td>Rutin</td><td>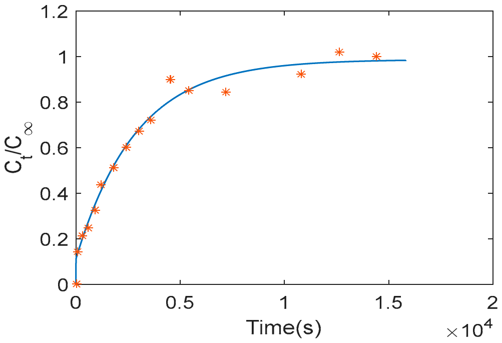
</td><td>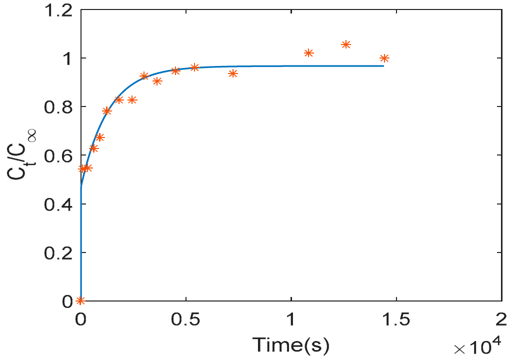
</td><td>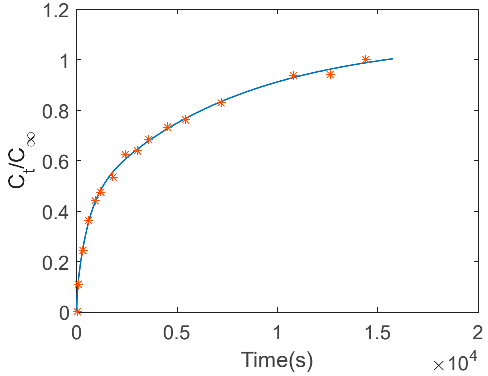
</td></tr>
<tr><td>Coumarin</td><td>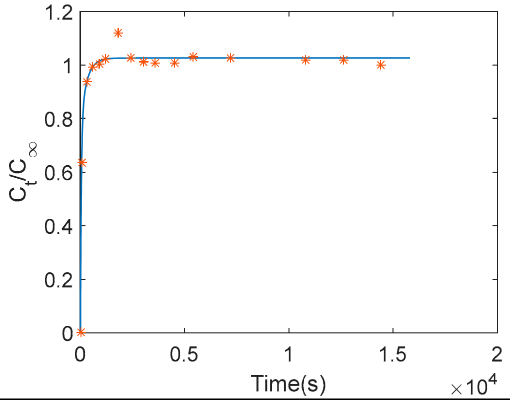
</td><td>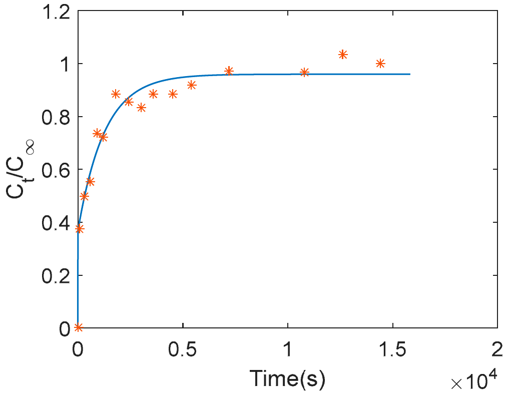
</td><td></td></tr>
<tr><td>Gallic Acid</td><td>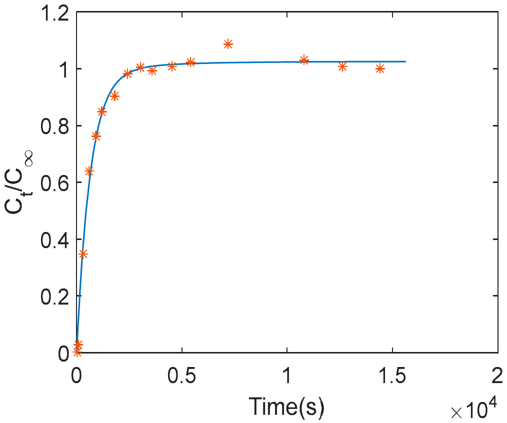
</td><td></td><td>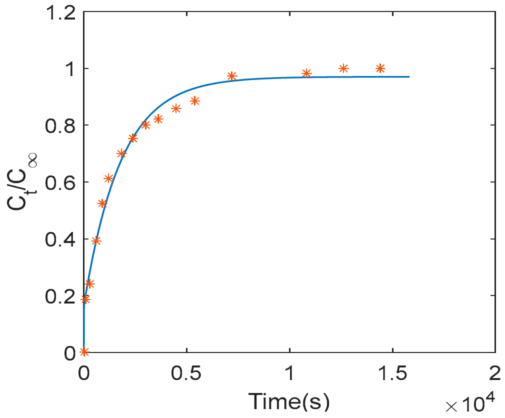
</td></tr>
</table>
